# Closing the circle of germline and stem cells: the Primordial Stem Cell hypothesis

**DOI:** 10.1186/2041-9139-4-2

**Published:** 2013-01-08

**Authors:** Jordi Solana

**Affiliations:** 1 Laboratory of Systems Biology of Gene Regulatory Elements, Max-Delbrück-Center for Molecular Medicine, Berlin, Germany

**Keywords:** Weismann barrier, Germ plasm, Nuage, Epigenesis, Preformation, Germline, Stem cells, Germ cells, Regeneration, Neoblasts

## Abstract

**Background:**

Germline determination is believed to occur by either preformation or epigenesis. Animals that undergo germ cell specification by preformation have a continuous germline. However, animals with germline determination by epigenesis have a discontinuous germline, with somatic cells intercalated. This vision is contrary to August Weismann’s Germ Plasm Theory and has led to several controversies. Recent data from metazoans as diverse as planarians, annelids and sea urchins reveal the presence of pluripotent stem cell populations that express germ plasm components, despite being considered to be somatic. These data also show that germ plasm is continuous in some of these animals, despite their discontinuous germline.

**Presentation of the hypothesis:**

Here, based on recent molecular data on germ plasm components, I revise the germline concept. I introduce the concept of primordial stem cells, which are evolutionarily conserved stem cells that carry germ plasm components from the zygote to the germ cells. These cells, delineated by the classic concept of the Weismann barrier, can contribute to different extents to somatic tissues or be present in a rudimentary state. The primordial stem cells are a part of the germline that can drive asexual reproduction.

**Testing the hypothesis:**

Molecular information on the expression of germ plasm components is needed during early development of non-classic model organisms, with special attention to those capable of undergoing asexual reproduction and regeneration. The cell lineage of germ plasm component-containing cells will also shed light on their position with respect to the Weismann barrier. This information will help in understanding the germline and its associated stem cells across metazoan phylogeny.

**Implications of the hypothesis:**

This revision of the germline concept explains the extensive similarities observed among stem cells and germline cells in a wide variety of animals, and predicts the expression of germ plasm components in many others. The life history of these animals can be simply explained by changes in the extent of self-renewal, proliferation and developmental potential of the primordial stem cells. The inclusion of the primordial stem cells as a part of the germline, therefore, solves many controversies and provides a continuous germline, just as originally envisaged by August Weismann.

## Background

The concept that the germline is distinct from the soma was introduced in the late 19th century by August Weismann [1]. In his Germ Plasm Theory he emphasized the continuity of the germline, which is both totipotent and immortal. The germ plasm, a collection of genetic determinants found in egg cells (Table 1), is inherited by the cells that will later give rise to the gametes of the new organism. Thus, in Weismann’s theory, the germ plasm is present in a continuous line of cells that expands from the zygote of one generation to the zygote of the next generation. This line of cells is known as the germline (Table 1). The germline is capable of giving rise to all somatic cells, while it cannot be affected by them. The concept of the so-called Weismann barrier derives from this vision, stating that hereditary information can transit from the germline to the soma, but not *vice versa* and, therefore, the germline is not affected by any acquired mutation in the somatic cells (Table 1).

**Table 1:** Glossary of terms

Germline	Translated from the German ‘*Keimbahn*’, also translated sometimes as ‘germ track’; it is the line of cells that carry the genetic material from one generation to the next
Germ plasm	A collection of determinants often found in the oocyte of several animals and which are often inherited by the germline
Nuage	A wider term (literally ‘cloud’ in French) referring to the electron-dense perinuclear material or granules often found in multipotent and germline cells and whose composition is similar to the germ plasm. This material is known by many different names in different organisms: polar granules in *Drosophila melanogaster*, P granules in *Caenorhabditis elegans*, chromatoid body and intermitochondrial cement in mammals, chromatoid bodies in planarians, Balbiani bodies in many vertebrates and invertebrates, or germ granules in a more generalized way
Germline Multipotency Program (GMP)	A term recently coined by Juliano and co-workers and referring to a collection of genes often expressed in nuage-containing multipotent cells and germline cells. Composed of genes known to be involved in germline determination, germ plasm and nuage assembly, these genes are often also found in multipotent or pluripotent cells. Examples of these are *vasa*, *nanos*, *piwi*, *tudor*, *pumilio* and *bruno* genes, for instance
Primordial Germ Cells (PGCs)	The first cells in the developing germline to have only germ potential. They undergo mitotic expansion and later in development populate the gonads to give rise to germ cells
Germ Cells (GCs)	In a sexually-reproducing animal, the cells that give rise to the gametes in the gonads
Preformation	The process of germ cell specification that proceeds via zygotic maternally-deposited germ plasm components which are selectively inherited by germline cells. The specification signal is therefore intrinsic
Epigenesis	The process of germ cell specification that proceeds via signals that segregate a population of cells from a multipotent or pluripotent group of cells. The specification signal is, therefore, extrinsic
Weismann barrier	Derived from August Weismann’s theory that genetic information only flows from germline cells to somatic cells, and it cannot flow from somatic cells to the germline. In other words, changes in the germline genetic information can affect somatic tissues and can be passed on to the next generation, while somatic mutation can affect neither the germline nor the forthcoming generations

**Figure 1:**
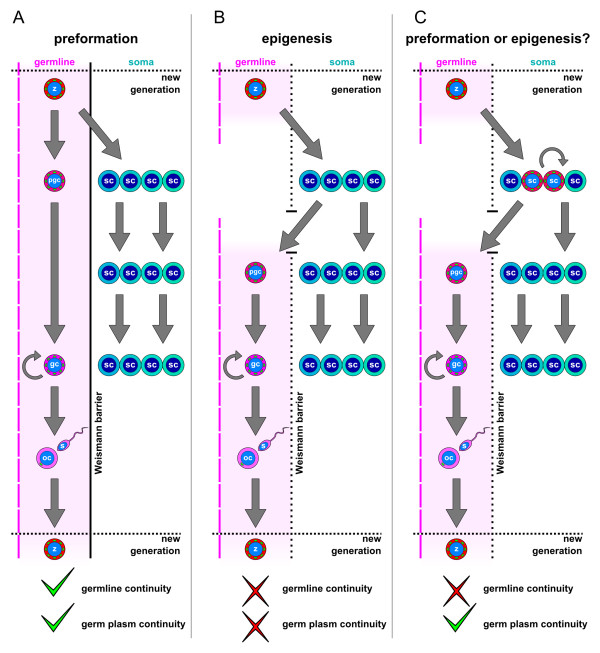
**The classical model of germline determination and its controversies.** (**A**) Germline determination by preformation. The germ plasm present in the zygote is inherited by the primordial germ cells (PGCs) and not by the rest of the somatic cells derived from it. The PGCs give rise to germ cells (GCs) and these in turn to sperm and oocytes. Somatic cells cannot affect the germline, and, therefore, the Weismann barrier can be easily imagined in this model. Both germline continuity and germ plasm continuity are observed. (**B**) Germline determination by epigenesis. The zygote gives rise only to somatic cells, from which a subpopulation is specified by epigenetic signals to become the PGCs. The Weismann barrier is, therefore, broken by these somatic cells, and neither the germline nor the germ plasm is continuous. (**C**) In animals classically thought to follow the epigenesis model as diverse as annelids and sea urchins germ plasm components are found in the zygote and inherited by cells with both somatic and germ potential. These cells give rise to the PGCs but also to somatic tissues, and often have stem cell-like properties. The Weismann barrier is broken by these cells, since they are classically considered to be somatic. However, even though the germline is considered to be discontinuous, germ plasm continuity can be observed flowing from the zygote to these cells and forth to the PGCs. (**A**-**C**) Germ plasm component expression is depicted in red-magenta colors and green dots. z, zygote; pgc, primordial germ cells; gc, germ cells; oc, oocyte; sc, somatic cell.

Later studies on the embryology of different model organisms revealed the existence of metazoans to which the concept of germline continuity can be hardly applied. Ultimately, this led to the proposal that two different paths of germline specification exist: preformation and epigenesis (Table 1) [2]. The organisms that specify their germline by preformation (Figure 1A) present germ plasm components in their zygotes which are inherited by the primordial germ cells (PGCs, Table 1) first and then by the germ cells (GCs, Table 1). Therefore, both germline continuity and germ plasm continuity are observed in this model, as proposed by Weismann. The Weismann barrier (Figure 1A) can be easily imagined in these organisms, since any mutation in their somatic tissue will eventually disappear with the death of the somatic cell or ultimately with the death of the organism. The germline will, therefore, stay clear of somatic mutations. However, the majority of metazoans are believed to specify their germline cells by epigenesis (Figure 1B), a process whereby epigenetic signals specify a group of somatic cells, sometimes called the presumptive primordial germ cells (pPGCs), to become the germline [2], of which the so-called PGCs are the first cells. In this model, the continuity of the germline is broken in early embryonic development, as somatic cells that exist after the zygote stage are induced to form the germline of the organism (see [3]). Therefore, in this mode of development somatic mutations can trespass into the germline, eliminating the concept of the Weismann barrier in these organisms.

It has become clear that the germ plasm is composed of several proteins and mRNAs, including for instance Vasa, Nanos and Piwi, which are typically organized in electron-dense granules. These granules, often known by different names in different species but collectively known as nuage (Table 1) and their molecular components are found in the egg cells and the zygotes of many species, but also in the germ cells. Only recently, the study of the expression patterns of several germline components in non-classical model organisms (collectively reviewed in [4]) has added some extra controversy to this old question [3]. In particular, several germ plasm components have been found to be expressed in tissues and cells classically considered to be somatic, such as planarian neoblasts [5-8], the mesodermal posterior growth zone (MPGZ) in polychaetes [9,10], the interstitial stem cells (I-cells) of hydrozoan cnidarians [11-13], and the small micromere lineage in echinoderm embryos [14]. Together, these findings have led to the proposal of a conserved germline multipotency program (GMP, Table 1) [4] that operates in both germline cells and somatic multipotent cells [15]. In most of these animals the germ plasm seems to be continuous, flowing from the zygote to these multipotent cells and forth to the GCs (Figure 1C). The classical model of preformation and epigenesis is, therefore, challenged, with a growing number of animals in which a germ plasm continuity is observed at the molecular level [8-10,13,14,16-23], despite their classification as epigenesis (Figure 1C). The specification of these multipotent cells and the germline often occurs in parallel, as has been shown in *Platynereis dumerilii*[10,19], generating controversy on the mechanism of this specification.

The root of the controversy, however, can be traced back to the original definition of the germline. This definition never considered the wide variety of metazoans capable of undergoing asexual reproduction and with high regenerative capacities. Here, I revisit the original definition of germline and the Weismann barrier and revise accordingly the concept of germline in order to include asexually reproducing metazoans. Out of this revision, and after careful inspection of the available data, a model that accurately describes the germline specification of most described organisms emerges. The existence of a highly conserved population of zygote-derived multipotent cells is deduced from this model. I call these cells the primordial stem cells (PriSCs), which have their representatives in planarian neoblasts, the 4d lineage of annelids and molluscs, the small micromere lineage of sea urchins, the inner cell mass (ICM) of the mammalian embryos, and the precursors of the PGCs of model organisms whose germline specification has been classically described to proceed by preformation. Only a few characteristics of these PriSCs change in each of the classes here described, hence providing an evolutionarily dynamic framework that explains the multiple transitions observed, even within animals belonging to the same phylum, across evolution.

## Presentation of the hypothesis

### The germline concept and the Weisman barrier in asexually reproducing animals: planarians specify their germline from neoblast cells

Freshwater planarians are free living platyhelminthes that often undergo both sexual and asexual reproduction by fission and present remarkable regenerative capacities. This is largely based on a population of undifferentiated stem cells present in their adult stage, the so-called neoblasts [24-26]. Neoblast cells present striking similarities to GCs, both at the morphological and molecular level. The presence within these cells of a kind of perinuclear RNA granules, called chromatoid bodies, constitutes a remarkable similarity with the germline granules, collectively known as nuage, that are known to be present in the germline of almost any studied metazoan [27,28]. The molecular components of the germline granules, which constitute what has been called the GMP [4] and include, for instance, Piwi, Vasa, and Tudor proteins, are also expressed in planarian neoblasts [5-8]. Despite these similarities, neoblasts are capable of giving rise to all somatic tissues [29].

It is currently unknown if or how planarians specify their germline during embryonic development, basically due to the inaccessibility of the highly derived triclad embryonic development [16,30] (reviewed in [31]). Briefly, the zygote of *Schmidtea polychroa* cleaves into blastomeres that are dissociated and embedded in a yolk syncytium. Embryonic cells (blastomere derivatives) proliferate in this syncytium and also give rise to the somatic structures of what is believed to be a cryptic larval stage [16,30]. Later, a wave of differentiation of the embryonic cells gives rise to the adult tissues. The first cells resembling neoblasts arise after this stage [8]. The embryo hatches as a juvenile worm with all adult structures except the gonads, which develop later. PGCs, however, have been detected in late planarian embryos [32], although this point remains controversial [33].

However, it has been shown that, regardless of their origin, neoblast cells always retain the potential of regenerating GCs when lost [32-34] and, hence, their specification has been considered to proceed by epigenesis [2]. The few existing data about the embryonic expression patterns of GMP genes point, however, to a very early expression of these genes in cleaving blastomeres, as has been described for the Tudor homologue Spoltud-1 [8], which is also localized in nuage*-*like granules present in these cells [16]. Embryonic germline specification, therefore, occurs most likely from a population of GMP-positive neoblast-like pluripotent stem cells. Therefore, the germ plasm, understood as both the presence of nuage material and GMP components, seems to be continuous in planarians.

**Figure 2:**
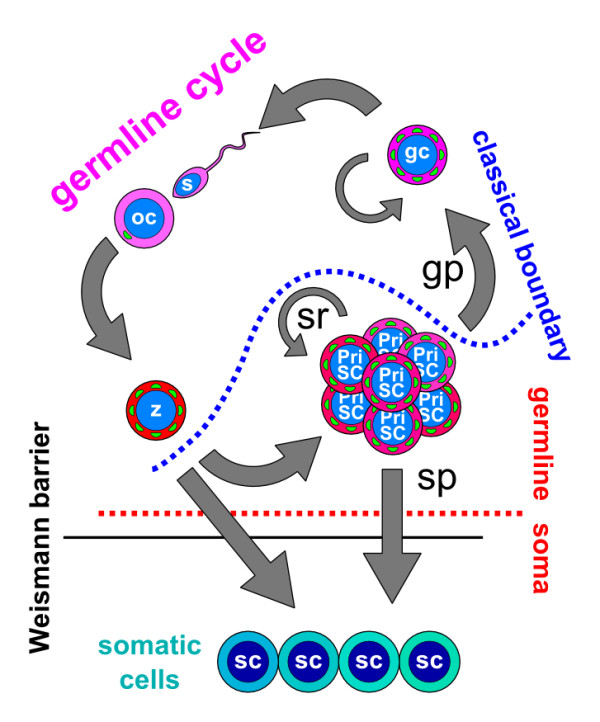
**The germline cycle in freshwater planarians.** Freshwater planarians possess a population of stem cells, the so called neoblasts, which represents the PriSCs in these organisms (PriSC). Neoblasts are able to give rise to the GCs (gc) and to somatic cells (sc). GCs give rise to oocytes (oc) and sperm (s), which jointly give rise to the zygote (z). The zygote gives rise to both somatic cells and the PriSCs. The planarian PriSCs have unlimited self-renewal (sr) and both germ potential (gp) and somatic potential (sp). Green dots represent the presence of nuage granules and germ plasm components. The dotted blue line represents the position of the germ-to-soma boundary, as classically understood. The dotted red line represents the proposed position of the germ-to-soma boundary as postulated in the Primordial Stem Cells hypothesis, which coincides with the Weismann barrier (solid black line) in freshwater planarians. PriSCs, primordial stem cells.

**Figure 3:**
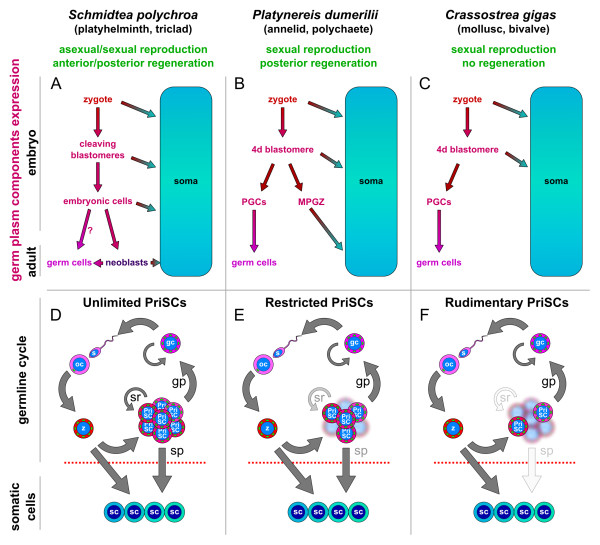
**Germ plasm components expression in lophotrochozoans and their germline cycles.** (**A**-**C**) Schematic of *Schmidtea polychroa* (**A**), *Platynereis dumerilii* (**B**) and *Crassostrea gigas* (**C**). (**A**) The cleaving blastomeres of *S. polychroa* express germ plasm components and likely give rise to the embryonic cells. These cells are believed to give rise to the neoblasts, but also to GCs and somatic tissues. (**B**) A putative germ plasm is found in the zygote of *P. dumerilii* and inherited by the 4d blastomere, which generates the PGCs but also the MPGZ, a germ plasm component-containing proliferative tissue with somatic potential (**C**) The early embryonic development of *C. gigas* is similar to that of *P. dumerilii*. However, only 2 cells derived from the 4d blastomere still show germ plasm components: they are believed to be the PGCs and become quiescent until later stages. (**D**-**F**) Modes of germline cycle in *S. polychroa* (**D**), *P. dumerilii* (**E**) and *C. gigas* (**F**). (**D**) Unlimited PriSCs: the zygote in *S. polychroa* gives rise to a population of stem cells (the PriSCs) with self-renewal (sr) and both somatic (sp) and germ potential (gp). (**E**) Restricted PriSCs: In *P. dumerilii* the 4d lineage is a population of pluripotent stem cells with both somatic (sp) and germ potential (gp) with more restricted self-renewal. (**F**) Rudimentary PriSCs: The germ plasm-containing cells of *C. gigas* only retain dual germ/soma potential for a few divisions and only the PGCs retain germ plasm components. Dotted red lines depict the proposed germ-to-soma boundary. GCs, germ cells; MPGZ, mesodermal posterior growth zone; PGCs, primordial germ cells; PriSCs, primordial stem cells.

The specification of the germline in planarians, then, likely follows the model proposed in Figure 2. A population of GMP-positive nuage-containing undifferentiated and pluripotent stem cells is derived from the early blastomere divisions, and the GCs are singled out from it either during embryonic development or later. This embryonic population is maintained in the adult organism, the neoblasts, and is capable of repeatedly regenerating the GCs (Figure 3A). The GCs of the adult are responsible for sexual reproduction but asexual reproduction (and regeneration) is driven by the population of neoblast cells.

The classical germ-to-soma boundary in planarians is indicated by the dotted blue line in Figure 2. Neoblast cells have been classically considered to be somatic stem cells, since they give rise to somatic tissues. Therefore, the germline only contains the GCs and their derivatives, the gametes and the zygote. However, while the germ plasm, evidenced by GMP components and nuage, seems to be continuous in planarians, this line breaks the continuity of the germline and the germline cycle here described, and I believe that this breakage is the root of the controversies explained above. Therefore, I propose a revision of the germline concept, in order to include the neoblasts, as indicated by the dotted red line in Figure 2. This proposal is made on the following basis. First, neoblasts resemble GCs both morphologically and at the molecular level and, therefore, might function in a similar way. Second, neoblasts have the ability to drive reproductive processes; both by asexual reproduction and by generating GCs. Third, the ability to give rise to somatic tissue cannot be used to exclude neoblast cells from the germline, since they share this property with the zygote itself. Lastly, and importantly, this definition would comply with the concept of the Weismann barrier (Figure 2, black line). The hereditary information can flow from both GCs and neoblast cells to somatic tissues, but it cannot flow in the opposite direction. For instance, mutations occurring in neoblast cells can be incorporated in both the soma (by means of cell differentiation, asexual reproduction and germline regeneration) and in the GCs (since these can be regenerated from neoblast cells). On the contrary, mutations occurring in differentiated somatic cells will be lost without possibly being incorporated into either neoblasts or GCs. The old concept of a Weismann barrier is, therefore, useful in order to delineate the germline.

The idea of neoblasts constituting a kind of asexual germline that operates during asexual division and by regenerating the sexual germline is new. At first sight, it seems counter-intuitive, due to the fact that asexually reproducing animals have been traditionally understudied, and the germline concept has only been associated with sexual reproduction. However, there is little doubt in the current literature that neoblasts are the cell lineage that contributes the genetic information to forthcoming generations of asexually dividing planarians by driving the regeneration and cell turnover of animals after fission. This is at least true for the subset of neoblasts (cNeoblasts) that have been experimentally demonstrated to be pluripotent, clonogenic and capable of restoring regeneration in lethally irradiated hosts [29,35]. In these experiments, lethally irradiated sexual strain worms were rescued by the implantation of a single neoblast and completely transformed to the genotype of the asexual donor strain, showing that neoblasts contribute their genetic information to the soma [29]. The opposite experiment, with a sexual donor strain, would demonstrate whether cNeoblasts can restore all tissues including the germline. Further work is needed in order to determine the level of heterogeneity of the neoblast population (see [25]) and, particularly, what is the proportion of cNeoblasts in this population [29,35,36].

Another line of argumentation for such a revision of the germline conception lies in the function of nuage particles and the GMP. Little is known about this matter, although it is clear that *piwi* genes and related piwi-interacting RNAs (piRNAs), for instance, function in the control of selfish transposable elements [37-39]. The inclusion of neoblasts as a full member of the germline cycle in freshwater planarians would explain the need for such cellular machinery, which is not essential in somatic cells due to the Weismann barrier limitation, but is needed in neoblasts since they propagate the genome during asexual reproduction and permanently retain the ability to give rise to GCs.

Molecular data from other platyhelminthes indicate that the expression of GMP genes in neoblast cells and GCs is widespread among the members of the phylum. For instance, *piwi* and *Vasa* homologues are expressed in both GCs and neoblasts in *Macrostomum lignano*[40,41]. However, the questions of whether these cells both derive from embryonic GMP-positive populations and the presence of germ plasm components in the zygote itself remain to be addressed. Future lines of research should try to clarify these questions by studying the localization of GMP components coupled with lineage tracing studies during the embryology of basal platyhelminthes [42], such as *Macrostomum lignano* or the polyclad *Hoploplana inquilina*, since their spiral cleavage is more amenable to lineage tracing studies [43,44].

### Widespread presence of stem cells in basal metazoans

Molecular studies on the expression of GMP components in cnidarians have revealed as well the expression of these genes in undifferentiated stem cells and their propagation through a continuous germ plasm. The *vasa* transcript is missing in the zygote of the hydroid *Hydractinia echinata*, but Vasa protein is present in a specialized region of the zygote, implying it is a kind of germ plasm [13]. The adult stage of *H. echinata* and other cnidarians also contains a population of I-cells which are capable of giving rise to both somatic tissues and gametes [45]. Furthermore, it has been shown in the hydrozoan *Clytia hemisphaerica* that GMP components are also localized as a germ plasm in the oocyte and are inherited by I-cells [18]. Recently, RNA-seq molecular profiling of gene expression has been achieved in planarian neoblasts [46-49] and cnidarian I-cells [50], revealing extensive similarities in their gene expression patterns. Therefore, cnidarian I-cells resemble neoblast cells in their dual somatic and germ potential, their expression of GMP markers and their sustained perdurance over the adult phase. Hence, cnidarians also likely follow the model proposed in Figure 2.

It has been reported recently that both archeocyte and choanocyte cells express *piwi* homologues in the freshwater sponge *Ephydatia fluviatilis*[51]. Archeocytes are undifferentiated stem cells which have the ability to differentiate to at least several of the differentiated cell types that constitute these organisms (reviewed in [52]), and they progressively lose the expression of *piwi* as the differentiation process takes place. However, it has been reported that archeocytes can undergo differentiation to choanocytes as well, and the expression of *piwi* is sustained during this process [51]. Choanocytes, remarkably, have the ability to give rise to gametes, mostly sperm. Interestingly, choanocytes are very similar at the morphological and functional level to the unicellular choanoflagellates, believed to be the sister group of all metazoans and, therefore, their closest unicellular relative.

### Pluripotent cells from spiralian embryos

Spiralians are a group of lophotrochozoans that share the archetypal spiral cleavage [53], making evolutionary comparisons easy and illustrative. However, their respective embryonic development programs differ after spiral cleavage. For instance, a Müller’s larva is formed in some polyclads [42]. Trochozoans, however, form a trochophore larva, which is shared by groups as different as annelids and molluscs [54], the most widespread and well-studied trochozoan groups, and others [55]. Annelidian trochophore larvae grow posteriorly through the MPGZ, giving rise to the segments that will shape the adult animal. Molluscan trochophore larvae are coiled instead to shape another kind of larva, the veliger larva, from which the adult animal develops.

A broad conservation of cellular fates has been observed in different spiralian embryos. For example, the 4d blastomere of spiralian embryos (the so-called ‘mesentoblast’) is known to give rise to endomesoderm in most examples [53,56,57]. The germline is also known to arise consistently from this particular blastomere in many spiralians [9,10,17,19,53,58-64] (Figure 3B-C). Cell lineage tracing studies, however, are not possible in every organism, and this question is, therefore, difficult to address rigorously.

One beautiful and illustrative example of a combination of cell lineage tracing and molecular expression profiling of germ plasm components is that carried out by Rebscher and co-workers in the annelid species *Platynereis dumerilii*[10,19] (for an account of *P. dumerilii* embryonic development see [65]). In *P. dumerilii* Vasa protein is localized to a specific region of the oocyte and, upon the beginning of development, it becomes progressively restricted to the D quadrant and highly enriched in the 4d blastomere (Figure 3B). This blastomere further divides to give rise to four cells that will later migrate anteriorly and give rise to the GCs, the PGCs of *P. dumerilii*, and to a highly proliferative area, the MPGZ, which will give rise to somatic tissue exclusively. Both the PGCs and the MPGZ express Vasa and other GMP components [10,19]. These characteristics establish a striking parallelism between the 4d lineage of *P. dumerilii*, understood as including both the germ plasm-containing precursors and descendants of the 4d blastomere, and planarian neoblasts in their dual somatic and germ potential and the presence of germ plasm components (Figure 3D). Essentially, annelidian 4d lineage cells and planarian neoblasts behave very similarly and their only difference is their final fate and developmental potential (Figure 3E). In freshwater planarians, neoblasts are present throughout the adult stage; however, in annelid worms, it is not yet clear if multipotent GMP-positive cells are present in the adult. Nevertheless, the continuity of the germ plasm is still present in *P. dumerilii*, flowing from the zygote to the 4d blastomere and forth to GCs and MPGZ cells.

Studies in the annelid species *Capitella teleta* indicate that the expression of GMP components in this distant polychaete species [66] is similar to that of *P. dumerilii*[17] and that posterior regeneration observed in some annelid species is driven by GMP-positive cells [9]. However, whether these regenerative cells are derivatives of the MPGZ or express GMP components *de novo* is still a question.

The embryonic development of gastropod and bivalve molluscs also proceeds by spiral cleavage and the formation of a planktonic trochophore larva. The expression pattern of several GMP components has already been described during the embryonic development of several molluscs. The early embryonic expression of a *vasa* homologue in the oyster *Crassostrea gigas* was found to be similar to that of *P. dumerilii*[58]. The mRNA from the oyster *vasa* gene is found in the vegetal pole of oocytes and becomes progressively restricted to the 4d blastomere (Figure 3C). Then, the mRNA is detected in two cells, presumably two daughter cells of the 4d blastomere (Figure 3C). These two cells persist without further proliferation during the subsequent larval stages and are therefore believed to be the PGCs of *C. gigas.*

Similar patterns have been described in other mollusc species with some modifications. In the snail *Ilyanassa obsoleta* the mRNA of a *vasa* gene is broadly expressed in the early cleavages and progressively restricted to the 4d blastomere and its descendants. Later, however, the expression of *vasa* disappears and it is only seen later in the adult stage [64]. Similarly, a *nanos* transcript is also restricted to the 4d blastomere and its descendants in *I. obsoleta,* and its knock down has been shown to affect somatic cells [67]. In the mollusc *Haliotis asinina*, a more basal gastropod [68], both *vasa* and *nanos* mRNAs are expressed broadly in the zygote and become restricted later to the 4d blastomere. Their expression can be followed until the mesodermal bands of the trochophore larva, which likely give rise to somatic tissues and to the germline [69].

Therefore, some molluscs seem to follow a model similar to that of the annelid *P. dumerilii* (Figure 3E), but in contrast, the case of the mollusc *C. gigas* shows a more restricted fate for the embryonic GMP-positive cells (Figure 3F). The GMP-positive descendants of the 4d blastomere in *C. gigas* do not exhibit any self-renewing properties, nor do they seem to contribute to any somatic tissues, but only to the germline, and they can, therefore, be called PGCs. However, the parallelism with different species of closely related molluscs and annelids unveils that while the 4d blastomere only generates one germ plasm-containing cell type, believed to be the PGCs, in *C. gigas*, it generates populations of self-renewing, germ plasm-containing cells with somatic potential in other spiralians, such as the MPGZ in *P. dumerilii*. The model depicted for the mollusc *C. gigas* resembles what has been described for most organisms whose germline is specified by preformation (Figure 1A), including *Drosophila melanogaster*, *Caenorhabditis elegans* and *Xenopus laevis*, among others: a group of cells that are specified by preformation are set aside during embryonic development. Only a few divisions separate the zygote from the PGCs. The cells undergoing these divisions express GMP components and contribute to the soma. Through the comparison of the distinct embryonic developments of spiralian organisms, I propose that these cells can sometimes give rise to populations of stem cells with both somatic and germ potential and, in some animals, often with regenerative potential and asexual reproduction, these populations can even be present in the adult stage. I thus call these cells the primordial stem cells (PriSCs) and define them as a group of multipotent or pluripotent cells that can have mixed germ/soma potential and that are always intercalated between the zygote and the PGCs during embryonic development. I propose that the PriSCs are part of the germline cycle, and this fact explains the presence of germ plasm components in them. The inclusion of the PriSCs in the germline cycle solves the long-standing question of germ plasm continuity in diverse groups such as annelids and platyhelminthes. Unlike the pPGCs introduced earlier in the literature, the PriSCs can have unlimited self-renewal and be present in the adult stage. Also, the use of the Weismann barrier concept allows delineating the PriSCs as part of the germline, since mutations occurring in any of these cells are competent to trespass the GCs and to be incorporated in forthcoming generations by either sexual or sometimes asexual reproduction. For instance, mutations occurring in the 4d lineage of either annelids or molluscs, or in the neoblast cells of triclad flatworms can all be transmitted to the GCs of these animals and, therefore, to forthcoming generations, and this fact might explain the presence of germ plasm components in them.

### The primordial stem cells: three models of PriSCs in terms of developmental fate and self-renewal

I describe three models of the germline cycle, depending on the behavior and potential of PriSCs. In the first one (unlimited, Figure 3D), the PriSCs can continuously self-renew, even throughout the adult stages of the animal, and PriSCs always retain a mixed germ/soma potential. This model is likely followed by triclad planarians, but also by acoel worms, cnidarians, sponges, and other animals with high regenerative capabilities and the potential for asexual reproduction. In the second model (restricted, Figure 3E), the PriSCs retain a mixed germ/soma potential but their self-renewing capacities are limited. This model can be observed in *P. dumerilii* and other annelids and molluscs, for instance. In the third model (rudimentary, Figure 3F), the PriSCs derived from the zygote only exhibit somatic potential and self-renewing properties for a very limited number of divisions during early embryonic development. The mollusc *C. gigas* likely follows this model, as well as the organisms considered to specify their germline by preformation, such as *C. elegans*, *D. melanogaster* and *X. laevis*.

Although the idea of multipotent or pluripotent progenitors being the precursors of the germline has been proposed previously by others [2-4,15,25], the addition of an asexual germline role for some of these cells helps in clarifying the variability observed in their different fates and potentials. Previous attempts relied on the somatic nature of these cells, a vision that has led to controversies. Their inclusion in a germline cycle observed in many phylogenetically distant animals strongly argues for the inclusion of these cells, the PriSCs, in a continuous germline concept according to the Weismann Barrier.

### A case study of rudimentary PriSCs: *C. elegans* germline specification

**Figure 4:**
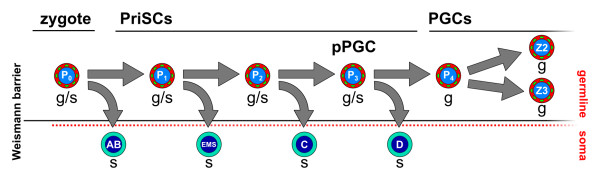
**The PriSCs of *Caenorhabditis elegans*.** Schematic of *C. elegans* early development. The zygote (P_0_) gives rise to the somatic AB cell and the series of germline blastomeres (P_1_, P_2_, P_3_ and P_4_), which retain the P granules (green dots). Each of them has both somatic and germ potential and divides to give rise to one somatic cell and another germline cell, except P_4_, which only has germ potential and can, therefore, be called PGC. P_4_ arises from the division of the presumptive primordial germ cell (P3, pPGC), and gives rise to the PGCs Z2 and Z3, which will remain silent during development and later proliferate to give rise to the GCs. Therefore, P_1_ to P_3_ are the rudimentary PriSCs of C. elegans. The sizes and shapes of cells are purely schematic and do not represent their actual shapes and relative sizes. PGCs, primordial germ cells; PriSCs, primordial stem cells.

Few animals have a cell lineage as well studied and understood as *C. elegans*[70,71]. The early divisions of the zygote (P_0_ cell, Figure 4) give rise to a series of germ plasm component-containing cells (the P lineage) which culminate in the P_4_ cell (Figure 4), which is the first PGC of *C. elegans*, with germ-only potential. P_4_ later divides in Z2 and Z3, which remain quiescent until hatching, and later proliferate to give rise to the adult germline. Each of the early divisions of the P lineage cells generates one somatic cell as well, therefore exhibiting mixed germ/soma potential. So does the zygote, which can be seen as the original PriSC in most animals. Therefore, the P lineage of *C. elegans* represents the PriSCs in this model, since all three cells (P_1_, P_2_ and P_3_) exhibit dual somatic and germ potential, and self-renewal for a very limited number of divisions.

Many organisms exhibit similar patterns in their early embryonic divisions. The generation of the 4d blastomere in molluscs for instance is reminiscent of this process of progressive restriction of germ plasm components into one cell, while generating somatic cells in each division. As has been discussed, however, other lineages of self-renewing germ plasm-containing cells can be generated in this process as well; such is the case of the MPGZ in *P. dumerilii*. These cells can undergo unlimited or limited self-renewal, regenerate the germ cells post-embryonically and drive asexual reproduction processes in many organisms.

The PriSC hypothesis provides, therefore, a very evolutionarily dynamic framework in which the regenerative capacities, the potential for asexual reproduction and the germline specification can be explained by the behavior of the PriSC population in each animal. Multiple transitions could be explained by only a few changes in the properties of PriSCs, and this could affect the life history of the organism. These changes, in the extent of self-renewal, proliferation or quiescence, and developmental potential, can be regulated by a small number of genes in an evolutionarily dynamic framework. A continuum of possibilities can be envisaged, enabling multiple transitions in different phyla. All three models proposed are present, for instance, in the spiralian group.

## Testing the hypothesis

### Widespread occurrence of the PriSCs in metazoans

Possible examples of the three models here described can be found widespread throughout the animal tree of life. For example, it has been reported that in the sea urchin *Strongylocentrotus purpuratus* Vasa and other GMP components accumulate in the small micromere lineage [4,14,72-74]. The small micromere lineage is a distinct population of cells that gives rise only to adult tissues, most likely including the germ cells [23,75]. Therefore, these cells, which express GMP components and have mixed germ/soma potential would constitute the PriSCs in sea urchin, likely following a restricted model similar to the evolutionarily distant *P. dumerilii.*

Most ecdysozoan and deuterostome animals reproduce sexually. However, some examples of asexual reproduction can be found among these phyla. For instance, ascidians, such as *Ciona intestinalis* reproduce sexually, and follow a typical example of germline specification by preformation [76-78], but closely related species of colonial ascidians can reproduce asexually. It has been reported that the colonial tunicates *Botryllus schlosseri* and *Botryllus primigenus* possess hemoblasts, which are undifferentiated stem cells that have both somatic and germ potential (reviewed in [79]). Some populations of hemoblasts express GMP components [80] and can specify the germline both at embryonic development and during asexual reproduction. Therefore, colonial ascidians likely follow the unlimited PriSCs model, while solitary ascidians such as *C. intestinalis* follow the rudimentary PriSCs model. However, further work is needed to demonstrate the existence of totipotent stem cells in colonial ascidians, as has been suggested [79].

However, little is known about the expression of germ plasm components in many organisms. The majority of phyla were considered to have either a mixture of preformation or epigenesis, or epigenesis only, with the only exception being the nematodes and the rotifers, which specify their germlines exclusively by preformation [2]. Molecular data on the expression of germ plasm components or GMP components is needed in all these phyla in order to elucidate and properly trace the PriSCs and the GCs. Also, for technical reasons, GMP components are more commonly detected on the basis of mRNA, not protein. This can lead to confusion; for instance, *vasa* mRNA was not detected in the zygote of the cnidarian *H. echinata*[13], but the protein was, as it is maternally supplied. As mRNA distribution does not always reflect protein distribution, cross-reactive or species-specific antibodies should be produced and used to immunolocalize GMP components in the early stages of development.

### Presence of germ plasm components in the primordial stem cells

The PriSC hypothesis postulates that GMP components and their morphological manifestation, nuage granules, are indeed part of a machinery to safeguard the genome and prevent the occurrence of mutation or transposition of selfish elements among others [38,39,81,82]. This machinery might, therefore, also be needed in cells that can propagate the genome to another individual, by either sexual or asexual reproduction. This fact explains its presence in pluripotent stem cells classically considered to be somatic, such as planarian neoblasts, echinoderm small micromere lineage cells and spiralian 4d lineage. Future studies should investigate if this is the case as well for other organisms. Combinations of lineage tracing studies and molecular labelling of GMP components will help elucidate the hypothetical presence of germ plasm components in zygote-derived germline-precursor pluripotent cells. Attention should be given to asexually-reproducing species belonging to mainly sexually-reproducing groups of animals, in order to understand if restricted or rudimentary PriSCs can evolutionarily revert to unlimited PriSCs. Such could be the case, for instance, of the colonial barnacle *Polyascus polygenea*, in which *vasa*-related mRNAs are expressed in somatic stem cells during the asexual reproduction process, in addition to GCs [83]. It would be important, then, to elucidate if these cells are derived from germ plasm component-containing cells during embryonic development and are, therefore, consistent with the PriSC hypothesis, or if the GMP component expression occurs *de novo* in somatic cells.

It is important to point out, however, that not all cells that can propagate the genome to future individuals might need GMP components. It is believed, for instance, that GMP components and nuage function are intimately linked to transcription, translation and regulation of gene expression processes in general [84]. PGCs, however, have been described as being quiescent in many organisms, from their specification to the onset of gametogenesis [85]. This fact would explain the reported disappearance of GMP components until the onset of gametogenesis in some molluscs, for instance.

Furthermore, many animals proceed through early developmental stages in a transcriptionally independent way [82,84,85]. Such is the case of *C. elegans*, in which transcription of zygotic genes in the germline starts in the Z2/Z3 cells [86]. P granules, the germ granules of *C. elegans*, a physical manifestation of the GMP, are present in the zygote and throughout early development. However, P granules in *C. elegans* are not likely to be functional during early development, since they are present at a cytoplasmic location. They only attach to the nuclear pores progressively in the P2 to P4 cells, before zygotic transcription starts [87]. Therefore, it can be hypothesized that their function in transcriptional and posttranscriptional control of gene expression might start at this point, and the P granules are only required as determinants in earlier stages. Such could be the case in several other organisms with transcriptionally inactive early embryogenesis.

It is also important to note that GMP components have been shown to play different roles in other somatic cell types, such as neurons [27,88,89]. The presence in neurons of a kind of RNA granule similar in composition to nuage granules might account for this presence and point to a similar function of these RNA granules. Common processes of stem cells, such as mitosis, can also be regulated by GMP components [73,90]. Furthermore, distinct separate subsets of GMP genes, such as *piwi* genes, might be needed in other cell types [91-93]: for instance, Piwi proteins are not only expressed in the germline of *D. melanogaster* but in the accompanying somatic cells, likely because transposons present in them can give rise to viral particles than can invade the germline [39]. In order to unravel this question, careful examination of gene expression patterns on a global scale is needed.

Since GMP genes can also be expressed in somatic tissues, it is important to understand the qualitative functional differences in germline and soma. For instance, although *piwi* expression is seen in *D. melanogaster* somatic cells, a much more intricate piRNA amplification cycle, the ping-pong cycle [94], occurs exclusively in the germline, with the involvement of the related proteins AGO3 and Aubergine, which are only found in the germline [38,39]. Therefore, qualitative differences in the gene expression patterns and the function of GMP components exist between germline and soma. The extent of these differences, however, remains to be explored. Thus, it would be interesting to test if the ping-pong cycle is truly germline exclusive in other animals and if it exists in PriSCs such as planarian neoblasts, for instance.

### The PriSCs enter the transcriptomic era

Several studies have already started to decipher in a global scale the expression profile of stem cells such as planarian neoblasts [46-49] and cnidarian I-cells [50]. Cnidarian I-cells were profiled along with the other, somatic, stem cells in the cnidarian *Hydra magnipapillata*. Interestingly, I-cells and neoblasts are closest at the gene expression level than neoblasts and any of the other two stem cell lineages of *H. magnipapillata*, suggesting that this resemblance could be due to the fact that they both represent lineages of PriSCs. It would, therefore, be interesting to investigate the potential contribution to forthcoming generations of the other two stem cell lineages of hydrozoans. No trace of somatic host cells was found by Wagner and co-workers in their key single neoblast transplantation experiment in planarians [29], but planarians lack other stem cell types of a somatic nature. Epidermal replacement is, for instance, undertaken by neoblast cells in triclad planarians and no mitoses are found in their epidermis. However, mitoses are found in the epidermis of many related groups [95], similar to hydrozoans.

Many organisms possess, then, true somatic stem cells. These stem cells, such as human epithelial or hematopoietic stem cells, never contribute to the next generation in sexually reproducing animals. Molecular profiling of these stem cells in many organisms and cross-comparison with the corresponding PriSCs will reveal if germ plasm components are needed simply for proliferation or stemness properties or are a direct consequence of the Weismann barrier limitation. Therefore, approaches to isolate or cultivate these stem cells are needed to perform global-scale gene expression surveys efficiently, both at the transcriptomic or the proteomic level.

## Implications of the hypothesis

### Developmental stages of primordial stem cells: preformation or epigenesis?

I have discussed the presence of a population of pluripotent stem cells, the PriSCs, during embryonic development. However, amid the myriad of transitions that the different cell populations in an embryo go through, all regulated by intrinsic or extrinsic signals, some of them must affect the PriSCs as well. Only the zygote/PriSCs and the PriSCs/GCs transitions are depicted in the model but, nevertheless, evidence from different model systems teaches us that this is an oversimplification. For instance, the zygote of *C. elegans* gives rise to a series of cells (the P lineage), each a product of a subsequent cell division (Figure 4). Each of these steps could potentially be intrinsically or extrinsically regulated (by a ‘preformation-like’ or ‘epigenesis-like’ kind of event). It is clear in *C. elegans* that the selective retention of P granules, an intrinsic signal, drives this process, rendering *C. elegans* as a model of preformation. The Z2 and Z3 cells become dormant until the onset of gametogenesis, which is another developmentally regulated transition.

Similarly, during the embryonic development of *P. dumerilii*, several transitions are also observed. First, Vasa protein is deposited in the zygote as an asymmetrically distributed maternal component, even though the mRNA distributes ubiquitously. Therefore, the first zygotic cleavages and the progressive restriction of germ plasm components to the 4d blastomere are a preformation event, despite the classification of germline specification in *P. dumerilii* as epigenesis. This fact was already noted by Rebscher and co-workers [10,19]. Subsequently, this one gives rise to the MPGZ cells and the PGCs.

In the triclad *S. polychroa*, different populations of GMP expressing cells are also detected [8,20]: first, embryonic cells in the early development and then, smaller sized cells which resemble neoblast cells. These cells likely give rise to the neoblast cells and to the GCs, denoted by the GC-specific expression of *nanos* in the related species *Schmidtea mediterranea*[32-34], although neoblasts retain the ability to give rise to GCs at any time, probably through an epigenetic event.

Therefore, the classification of germline specification in two different types, epigenesis and preformation, is deemed oversimplified and incomplete by the PriSC hypothesis. I propose that both the zygote/PriSCs and the PriSCs/GCs transitions, along with those occurring in the PriSCs themselves, can be by either preformation or epigenesis. Remarkably, animals following the rudimentary PriSCs model are mostly believed to specify their germlines by preformation. This fact is explained by the rudimentary state and fate restriction of the PriSCs in these animals.

### Germ plasm continuity revisited

The PriSC hypothesis states that even though the continuity of the germline is considered to be interrupted in animals following epigenesis as a means of germline specification, there is still a continuity of the germ plasm that flows from zygote to PriSCs and then to GCs. Therefore, if PriSCs are included in the germline definition, then the continuity of the germline is not interrupted either. A growing number of studies in recent years have pointed to a germ plasm continuity observed at the molecular level in a wide variety of animals, even those classically considered to specify their germlines by epigenesis [8-10,13,14,16-23].

However, it is difficult to translate the classical term ‘germ plasm’ into a 21st century molecular definition. The closest approximation would be the use of the GMP as the molecular circuitry that drives the germ plasm. The GMP might not be, nevertheless, a united and indivisible block, but a collection of molecular circuits [82]. Not all of them might be needed in all stages. It should also be noted that germ plasm or nuage granules can vary in shape, size and number from organism to organism. For instance, large electron-dense granules were found in the PGCs of the enigmatic Chaetognath worms [96]. In contrast, tiny *vasa*- and *nanos*-positive granules were recently described during the early cleavage steps of the cephalochordate *Branchiostoma floridae*[22]. Interestingly, this new finding shows that germline specification likely occurs by preformation in cephalochordates, although it was previously believed to occur by epigenesis, due to the previous lack of evidence regarding the presence of a germ plasm in oocytes and early embryos. Therefore, a close examination of GMP components could likely reveal the continuity of the germ plasm in other organisms that are believed to specify their germline by epigenesis.

### Mammalian PriSCs and epigenetic regulation

The PriSC hypothesis predicts that cells from the ICM and the epiblast, from which the PGCs are specified [97] are the mammalian PriSCs, and that they follow a restricted model (Figure 3E). Models similar to this have already been depicted in the literature (see for instance [98]). This notion poses, however, the caveat that GMP components are typically expressed in the PGCs of mammalian embryos but not before their specification [99]. However, several studies have already established parallels between ICM cells, or their *in vitro* derivatives, the embryonic stem cells (ESCs, [100,101]), and PGCs. Pluripotent stem cells, morphologically indistinguishable from ESCs were derived *in vitro* from PGCs without the use of reprogramming factors [102-104] long before these were used to reprogram non-pluripotent somatic cells into induced pluripotent stem cells (iPSCs [98,105]) for the first time [106]. It could be, therefore, that the known components of the GMP are just the tip of the iceberg of a wider genetic circuitry which would be present in both ESCs and PGCs. This wider GMP notion would include Oct4 as a master regulator in mammals [98]. Consistently, Oct4 is needed to reprogram somatic cells to iPSCs but not if PGCs or GCs are used, since they already express it. An *Oct4*-like gene has been found to be expressed in cnidarian I-cells and shown to reprogram cnidarian somatic cells [107,108]. Furthermore, several genes similar to *Oct4* and numerous Oct4 targets and regulators have been shown to be expressed in planarian neoblasts [47], implying that the network could be conserved in cnidarians and in planarians.

In support of the evolutionary conservation of this wider GMP circuitry, it has been recently reported that there is extensive conservation of the mechanisms that govern pluripotency in mammalian ESCs and planarian neoblasts [47]. It is, therefore, tantalizing to hypothesize that this close relationship is due to the fact that they are both representatives of the PriSCs in different organisms and that the shared components might also be part of the GMP molecular circuitry, even though the expression of classical germline components, such as Vasa, does not start until later in mammals when the PGCs are specified. Furthermore, recent molecular profiling of planarian neoblasts [36,46-49] and cnidarian stem cells [50] reveals that planarian neoblasts share molecular components, such as epigenetic regulators, with cnidarian I-cells, which are the progenitors of the cnidarian GCs and, therefore, the representatives of the PriSCs in these organisms. Interestingly, epigenetic regulation is in turn also shared by mammalian ESCs and their *in vivo* counterparts. Consistently, the other two cnidarian stem cell types, however, do not show such a prominent conservation of the expression of epigenetic regulators [50]. Therefore, these data point to epigenetic regulation as another part of an expanded GMP that drives pluripotency in the PriSCs of cnidarians, planarians and mammals.

### Inclusion of the primordial stem cells in the germline

**Figure 5:**
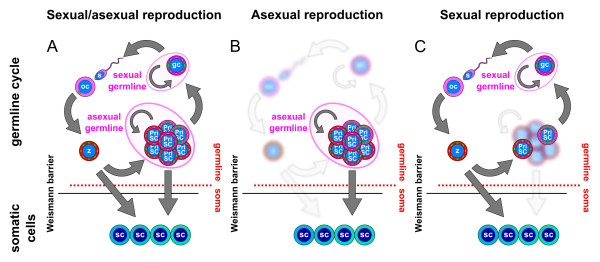
**Asexual and sexual germlines.** (**A**) Animals capable of both sexual and asexual reproduction possess an asexual germline and a sexual germline. (**B**) Animals that reproduce exclusively asexually have nonetheless an asexual germline, which resides in the PriSCs. (**C**) Animals that exclusively reproduce sexually have lost the self-renewing capacities and/or somatic potential of their PriSCs and, therefore, only a classical sexual germline is present. PriSCs, primordial stem cells.

As I have discussed here, the root of the multiple controversies regarding germline specification and its phylogeny comes from the exclusion of the PriSCs from the germline definition, while the precursors of PGCs are generally included in the organisms believed to specify their germline by preformation. The inclusion of all PriSCs in a more generalized germline concept would, therefore, eliminate the root of these controversies. Thus, animals that reproduce both sexually and asexually would have a sexual-type germline and an asexual-type germline (Figure 5A). The widespread occurrence of unlimited PriSCs in basal organisms such as sponges and cnidarians suggests that this was the model used by the first metazoans. With the inclusion of the PriSCs in the germline definition, purely asexual organisms, such as the asexual strains of freshwater planarians, would therefore have a germline, though an asexual-type one (Figure 5B). However, animals exclusively reproducing sexually lost their asexual-type germline due to the restriction in fate and self-renewal of their PriSCs (Figure 5C).

**Figure 6:**
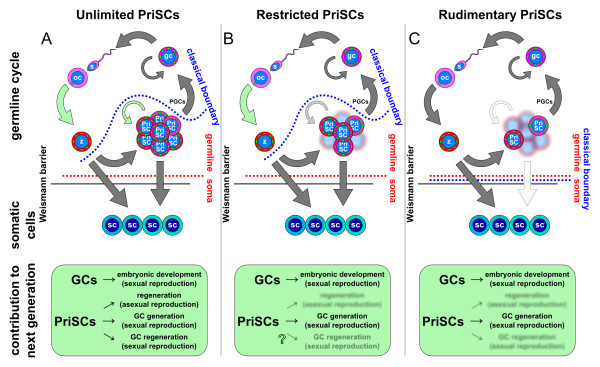
**The germ-to-soma boundary according to the classical view and the PriSC hypothesis.** (**A**-**C**) The dotted blue and red lines depict the classical and the proposed germ-to-soma boundary respectively. The black line depicts the Weismann barrier. Green arrows depict the origin of new generations. Green boxes indicate the source of contributions to forthcoming generations. (**A**) In animals with unlimited PriSCs these are classically considered to be somatic, breaking the germline continuity. The proposed germ-to-soma boundary makes both germline and germ plasm continuous and consistent with the Weismann barrier. New generations originate by sexual reproduction (large green arrow) or by asexual reproduction (small green arrow). The PriSCs contribute to forthcoming generations by enabling regeneration after asexual division, by embryonically generating the GCs or by regenerating GCs if these are lost. (**B**) In animals with restricted PriSCs the classical germ-to-soma boundary has to separate the PriSCs according to their germ or somatic potential and similarly breaks the germline continuity. The proposed germ-to-soma boundary again makes both germline and germ plasm continuous and consistent with the Weismann barrier. New generations originate only by sexual reproduction and the PriSCs only contribute to forthcoming generations by generating GCs. It is still unclear if PriSCs can regenerate GCs if lost (question mark). (**C**) In animals with rudimentary PriSCs the classical germ-to-soma boundary coincides with the proposed germ-to-soma boundary and the Weismann barrier, with both a continuous germline and germ plasm. New generations originate only by sexual reproduction and the PriSCs only contribute to forthcoming generations by generating PGCs. GCs, germ cells; PGCs, primordial germ cells; PriSCs, primordial stem cells.

The classical definition of germline in all animals has been biased by the consideration of sexually reproducing animals. As soon as non-conventional model systems with asexual reproduction or regenerative powers are considered the definition of germline starts to be controversial. For instance, planarian neoblasts, representatives of the unlimited PriSCs model, have classically been considered somatic stem cells since they give rise to somatic tissues. However, as I have discussed, the classical germ-to-soma boundary that excludes neoblasts from the germline (Figure 6A, blue line) also breaks germline continuity, while a germ plasm continuity is observed in these animals, flowing from the zygote to the PriSCs (embryonic cells and their derivatives, the neoblasts) and forth to the GCs. The PGCs, understood as the first cells with germ-only potential and their derivatives, would no longer be PriSCs and are, therefore, a distinct cell population. Similarly, controversy is posed by the classical germ-to-soma boundary as seen in animals that follow the restricted PriSCs model, such as *P. dumerilii*. In these animals this boundary (Figure 6B, blue line) separates the zygote from the 4d lineage and its derivative, the MPGZ. A similar exclusion of the small micromere lineage would occur if sea urchins are considered. However, the PriSCs of animals following the rudimentary PriSCs model have been classically considered to be part of the germline, despite their very limited, but existing, somatic potential (Figure 6C, blue line). This inclusion, while the closely related PriSCs with broader somatic potential are excluded, therefore, is at the root of the controversies explained above.

The proposed inclusion of the PriSCs in the germline concept is made on the basis that they can contribute in all animals to forthcoming generations. In the case of PriSCs of the unlimited model, exemplified by planarian neoblasts, they can do it by means of both asexual and sexual reproduction. Unlimited PriSCs can contribute to a next generation (indicated by green arrows): first, by being the cellular source during asexual reproduction; second, by generating germ cells; and third, by constantly regenerating them when lost (Figure 6A). It is also clear that PriSCs of the restricted model contribute to the next generation by generating the GCs during embryonic development (Figure 6B), just like the PriSCs of the rudimentary model (Figure 6C). However it remains unclear if restricted PriSCs can regenerate GCs when lost (Figure 6B). Many annelids, for example, can regenerate only posteriorly (while many others can regenerate both anteriorly and posteriorly and effectively reproduce asexually) [109,110]. It would be interesting to know if germ cells can be regenerated by cells from the PriSC lineage in these annelid species. These experiments would also be illustrative in other groups: similar to annelids, many triclads, for instance, do not possess the regenerative abilities of *S. mediterranea* and *Dugesia japonica*, the reference planarian model species, and hardly regenerate anterior fragments [111]. Therefore, they reproduce exclusively sexually. It would be interesting to know if neoblasts from these species can still regenerate GCs if these are lost.

## Conclusions

I define primordial stem cells (PriSCs) as highly conserved stem cells that are predicted to be intercalated between the zygote and the germline during embryonic development and that are delineated by the classical concept of the Weismann barrier. The behavior of the PriSCs, in terms of self-renewal and somatic and germ potential is related to the mode of reproduction and the regenerative capacities in each animal. In the most classic developmental model systems, the PriSCs are rudimentary and primarily give rise to the PGCs, along with a few somatic cells during early embryonic development. However, recent evidence reveals that the PriSCs in other animals are not so restricted and have different levels of self-renewal and somatic potential. The comparison of different animal embryonic developmental data reveals that proliferative, self-renewing, somatic precursor PriSCs are likely related to the more restricted precursors of the PGCs in more classic developmental organisms. Non-rudimentary PriSCs can also be restricted in fate or give rise to populations of stem cells with unlimited self-renewal. All kinds of PriSCs have similar gene expression patterns, as evidenced at the morphological level by the presence of nuage-like RNA granules. This presence can be explained by the Weismann barrier concept, since all kinds of PriSCs can effectively drive reproduction processes by means of sexual or asexual reproduction. Therefore, the machinery needed in GCs to carry out its genome keeping function is also needed in the PriSCs. The inclusion of the PriSCs in a more generalized germline concept, by the use of the Weismann barrier concept, resolves numerous controversies. This germline concept consists of a germline cycle with PriSCs and GCs, each capable of driving reproductive processes by means of asexual or sexual reproduction. Both germline continuity and germ plasm continuity are observed in the new germline concept, just as originally envisaged in the 19th century by August Weismann in his germ plasm theory.

## Abbreviations

ESC: Embryonic stem cell; GC: Germ cell; GMP: Germline multipotency program; I-cells: Interestitial cells; ICM: Inner cell mass; iPSC: Induced pluripotent stem cell; MPGZ: Mesodermal posterior growth zone; PGC: Primordial germ cell; PriSC: Primordial stem cells.

## Competing interests

The author declares that he has no competing interests.
